# Thoracic low grade glial neoplasm with concurrent H3 K27M and PTPN11 mutations

**DOI:** 10.1186/s40478-022-01340-9

**Published:** 2022-04-28

**Authors:** Michael G. Argenziano, Julia L. Furnari, Michael L. Miller, Yu Sun, Matei A. Banu, Justin A. Neira, Matija Snuderl, Jeffrey N. Bruce, Mary Welch, Paul McCormick, Peter Canoll

**Affiliations:** 1 Department of Neurological Surgery, Columbia University Irving Medical Center, New York, USA; 2 Columbia University Vagelos College of Physicians and Surgeons, New York, USA; 3 Department of Pathology and Cell Biology, Columbia University Irving Medical Center, 1130 St. Nicholas Avenue, New York, NY 10032 USA; 4 Department of Pathology, NYU Grossman School of Medicine, New York, USA; 5 Department of Neurology, Columbia University Irving Medical Center, New York, USA

**Keywords:** H3 K27M, Intramedullary tumor, Low grade glioma, PTPN11, MAPK signaling pathway

## Abstract

We present the case of a 41-year-old man who developed worsening mid-thoracic back pain and imaging revealed a well-circumscribed intramedullary tumor in the thoracic spinal cord. Subtotal resection was performed, and histopathological analysis showed a cytologically bland, minimally proliferative glial neoplasm. Sequencing revealed H3 K27M and an activating *PTPN11* mutation. Serial imaging revealed slow tumor regrowth over a three year period which prompted a second resection. The recurrent tumor displayed a similar low grade-appearing histology and harbored the same H3 K27M and *PTPN11* mutations as the primary. While the prognostic importance of isolated H3 K27M in spinal gliomas is well-known, the combination of these two mutations in spinal low grade glioma has not been previously reported. Importantly, PTPN11 is a component of the MAPK signaling pathway. Thus, as building evidence shows that low grade-appearing gliomas harboring H3 K27M mutations along with BRAF or FGFR1 mutations have a relatively more favorable course compared to isolated H3 K27M-mutant midline gliomas, the present case provides new evidence for the prognostic importance of activating mutations in other components of the MAPK signaling pathway. This case further highlights the importance of clinico-radio-pathologic correlation when incorporating evolving genetic data into the integrated diagnosis of rare neuroepithelial tumors.

**Supplementary Information:**

The online version contains supplementary material available at 10.1186/s40478-022-01340-9.

## Introduction

Mutations in histone genes resulting in H3 K27M are recurrently found in diffuse midline gliomas (DMGs), which are aggressive, high grade glial neoplasms that most commonly occur in children. The defining mutations result in an amino acid change of lysine to methionine at the 28th amino acid (p.K28M) of H3.1 or H3.3 proteins, encoded by *HIST1H3A* and *H3F3A* genes, respectively. The epigenetic reprogramming as a result of these histone mutations are thought to promote oncogenesis [1]. These mutations are most commonly seen in diffuse midline gliomas of the brainstem, thalamus and spinal cord, and in this context their presence carries a poor prognosis [2].

*PTPN11* is a proto-oncogene tightly linked to regulation of the RAS/MAP-Kinase pathway. Germline mutations in this gene are seen in Noonan Syndrome, an autosomal dominant multiple congenital anomaly syndrome associated with a wide range of malignancies, including neuroblastoma and leukemias [3]. Somatic mutations in *PTPN11* are linked to several solid tumors and hematologic malignancies [4]. While relatively rare, *PTPN11* mutations have also been detected in a variety of neuroepithelial tumors, including glioblastoma, oligodendroglioma, pediatric low grade gliomas (LGG), and rosette-forming glioneuronal tumors [5–10].

Here, we present a case of a 41-year-old male presenting with worsening mid-thoracic back pain, and diagnosed with a spinal neoplasm with low grade histopathological features and harboring both H3 K27M and *PTPN11* mutations.

## Case presentation

### Initial presentation

The patient, a 41-year-old man, initially presented with a six-week history of worsening mid-thoracic back pain about 16 months after being involved in a motor vehicle accident as a pedestrian. At the time of the motor vehicle accident, a full skeletal survey demonstrated a non-displaced proximal fibular fracture, nasal bone fractures, bilateral thigh hematomas and nonspecific mildly enlarged lymph nodes. The patient reported no back pain at the time of the accident, and limited spinal imaging demonstrated no acute fracture or subluxation in the cervical spine. The patient left the hospital within one day and recuperated at home with the assistance of outpatient physical therapy.

**Fig. 1:**
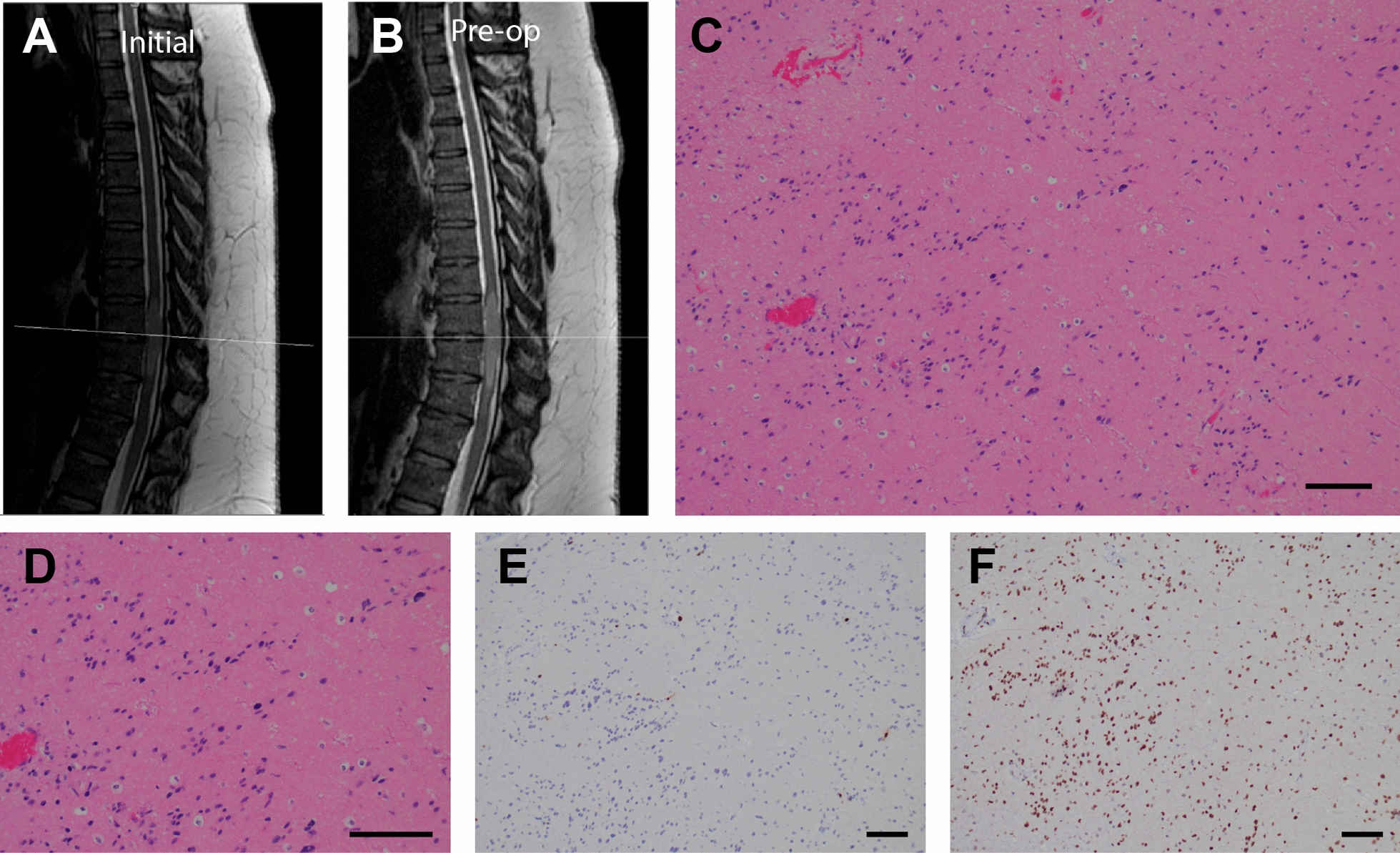
Initial presentation and histopathology of primary resection. **a, b** Initial sagittal T2 MRI (**a**) and repeat study 5 months later (**b**). **c, d** Hematoxylin and eosin (H&E)-stained sections revealed a glial neoplasm characterized by foci of alternating cellularity (**c**) and moderate pleomorphism (**d**). **e** Relatively rare cells were highlighted with KI67 immunostain. **f** The nuclei of the tumor cells were diffusely positive for mutant histone protein H3 K27M by immunostaining (for all histology panels, scale bar = 100 µm)

At presentation, the patient described the back pain as constant, dull, and non-radiating, and worsened with prolonged sitting. The patient reported no weakness, numbness, tingling or other focal neurological deficit. While physical therapy along with cyclobenzaprine and meloxicam provided temporary relief, the pain recurred and worsened which prompted imaging. Thoracic MRI revealed a well-circumscribed expansile intramedullary thoracic cord lesion, extending from T8–9 to T10–11 and measuring 5.3 cm in largest diameter, with no clear evidence of cord edema or cystic component (Fig. 1a).

On evaluation by neuro-oncology, the patient reported subjective numbness bilaterally in the anterior abdomen, inner thighs, and the soles of the feet. Complete neurological exam was unremarkable except for reduced vibration and proprioception in both toes. Following a short period of surveillance—in line with the patient’s preferences—repeat imaging 5 months later demonstrated significant interval growth of the intramedullary lesion (Fig. 1b). Although the neurologic exam was grossly unchanged, the patient felt as though their feet were dragging. Due to the interval growth in a short period of time, surgical resection was pursued. Intra-operatively, the tumor was mixed in with, and densely adherent to, the pia mater and vasculature, and was difficult to dissect from the underlying spinal cord. Thus, the tumor was internally debulked as safely as possible and a subtotal resection was achieved. At one month after the operation, the patient was improving slowly from a functional and symptomatic perspective, but had some residual numbness and spasticity in the right lower extremity, and post-operative MRI confirmed the presence of residual non-enhancing tumor.

### Primary pathology

Microscopic examination revealed a glial neoplasm with modestly pleomorphic nuclei which were loosely arranged into clusters and embedded within a course fibrillar background (Fig. 1c). A subset of the cells displayed smudgy chromatin suggestive of degenerative atypia (Fig. 1d), however no necrosis or mitotic activity was seen, and the KI67 labeling index was low (2.8%) (Fig. 1e). The tumor cells were also positive for GFAP and SOX2 (see Table 1). Within this small excision and in accord with the well-circumscribed radiographic description, the overall histology and immunophenotype were characteristic of a low grade glioma. Notably, an H3 K27M mutation was detected by immunohistochemistry (Fig. 1f). Genomic sequencing analysis (via targeted multiplexed sequencing on the Illumina MiSeq platform) confirmed the presence of an *H3F3A* mutation, and also revealed an activating *PTPN11* mutation (p.Q510H). While some histopathological similarities to subependymoma were noted, considering the genetic findings, the case was described as a “Subependymoma-like tumor, with H3 K27M mutation”.

**Table 1:** Immunostains from primary and recurrent resection

Marker	Primary	Recurrent
GFAP	Diffusely, strongly positive	Diffusely, strongly positive
CD44	Diffusely, strongly positive	–
PDGFRA	Weakly positive in a subset	–
SOX2	Positive in majority of tumor cells	Positive in majority of tumor cells
OLIG2	Positive in a subset	Positive in a subset (~ 20%)
SOX10	–	Positive in a subset (~ 20%)
IDH1 R132H	Negative	–
ATRX	Preserved	–
PTEN	Negative	–
TP53	Rare cells	–
H3 K27M	Positive	Positive
KI67	Up to 2.8%	Up to 2.4%

### Interval progression

**Fig. 2:**
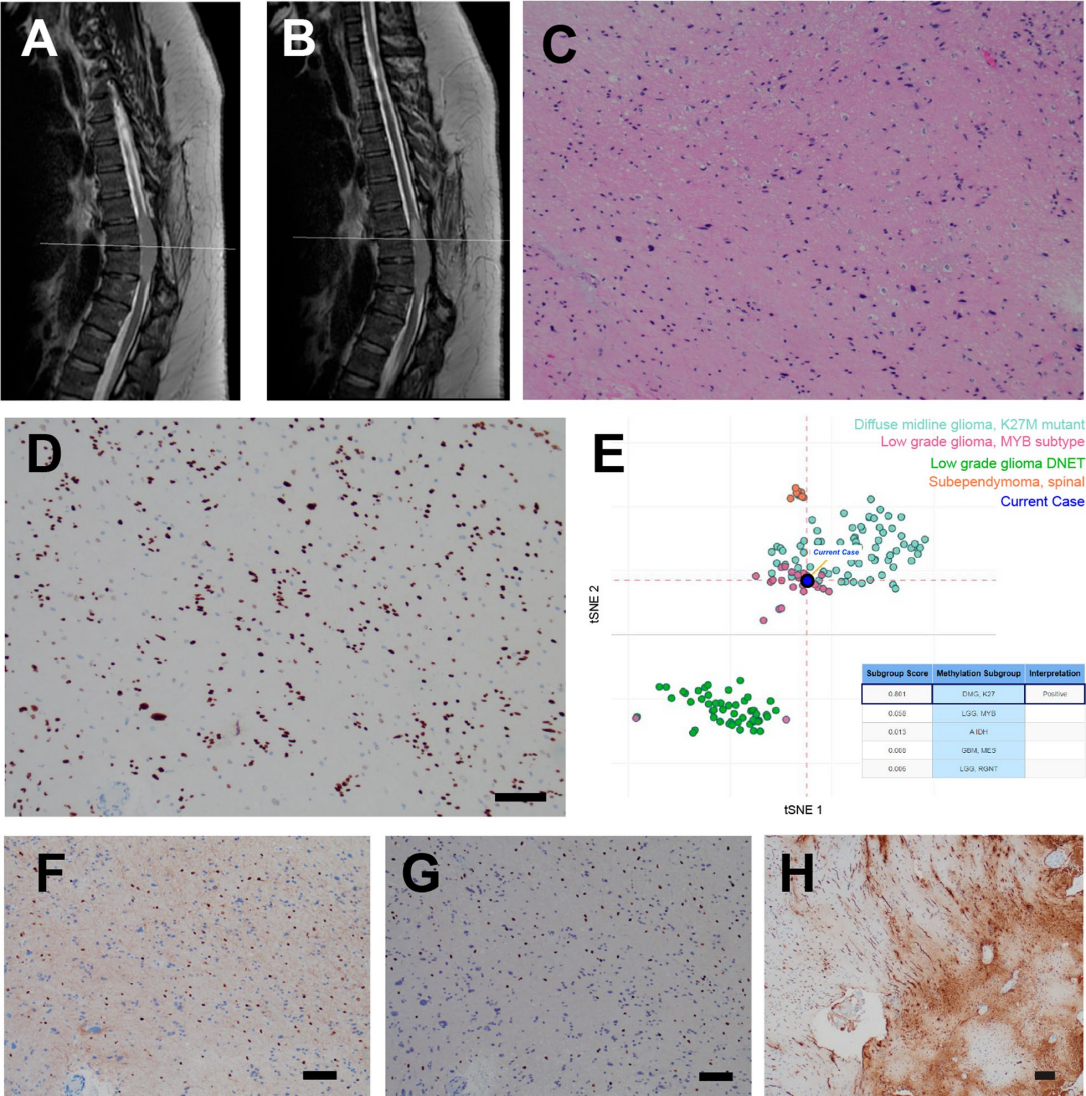
Post-operative surveillance, recurrence, and histopathology of second resection. **a, b** Sagittal T2 MRI 28 months (**a**) and 32 months (**b**) after initial resection. **c, d** Similar to the initial resection, hematoxylin and eosin (H&E)-stained sections revealed a glial neoplasm of biphasic cellularity (**c**) with immunohistochemical expression of H3 K27M (**d**) (for all panels, scale bar = 100 µm). **e** TSNE plot of methylation analysis, **f** SOX10 immunostain, **g** OLIG2 immunostain, **h** phospho-NF immunostain (for all histology panels, scale bar = 100 µm)

Over the next two years, the patient remained clinically stable, and no evidence of tumor growth was seen on initial imaging studies. That said, 28 months after the initial resection, imaging revealed interval growth (Fig. 2a) and repeat imaging 4 months thereafter revealed progression and a small focus of contrast-enhancement (Fig. 2b, Additonal file 1: Fig. 1A). After 10 months of further monitoring (42 total months after initial resection), the patient elected for repeat resection of the recurrent lesion. As with the prior surgery, it was difficult to achieve a safe complete margin, and an uncomplicated subtotal resection was performed (Additonal file 1: Fig. 1B, C).

### Pathology of recurrent tumor

Microscopic examination of the recurrent tumor revealed a low grade glial neoplasm similar to the original resection, with low to moderate cellularity (Fig. 2c) and low proliferative activity. In particular, no mitotic activity was seen and the KI67 labeling index was up to 2.4%. The immunoprofile of the recurrent tumor was similar to the initial resection, and the majority of cells were positive for the H3 K27M mutation (Fig. 2d). A subset of cells stained positive for OLIG2 (Fig. 2g) and SOX10 (Fig. 2f), with approximately 20% of cells staining positive for each of these markers in some areas of the tumor. Rosenthal fibers were identified in the recurrent tumor, however the affected regions were generally negative for the H3 K27M immunostain, and therefore likely corresponded to reactive piloid gliosis near the site of the prior resection. Focal invasion into the spinal parenchyma was confirmed with a phospho-NF immunostain (Fig. 2h). Molecular analysis of the recurrent tumor revealed the same genetic variants identified in primary resection, namely pathogenic mutations in *PTPN11* and *H3F3A*. Whole Genome DNA methylation profiling using clinically validated brain tumor classification [11] was performed and the tumor classified as diffuse midline glioma H3 K27M mutant with calibrated score 0.8 and the copy number analysis using *conumee* package showed flat copy number profile and unmethylated *MGMT* promoter. Interestingly, when DNA methylation data were analyzed using T-distributed Stochastic Neighborhood Embedding (tSNE) analysis, the tumor clustered between K27M and MYB low grade glioma clusters (Fig. 2e).

## Discussion

In this report, we present a case of a low grade glial neoplasm occurring in the thoracic spinal cord which harbored both H3 K27M (*H3F3A* p.K28M) and *PTPN11* (p.Q510H) mutations.

Neither H3 K27M nor PTPN11 mutations are frequently found in spinal tumors, and furthermore neither are classically associated with low grade gliomas. In comparison to patients with high grade glial neoplasms, those diagnosed with low grade gliomas, including spinal subependymomas, generally have a better prognosis. These low grade tumors rarely infiltrate the surrounding tissue, are slow growing, and are typically treated with surgical excision if symptomatic [13]. The H3 K27M mutation, however, is generally associated with high grade glial neoplasms. In diffuse midline gliomas, this molecular alteration carries a significantly worse prognosis. Earlier studies have suggested that the presence of a H3 K27M may override traditional histological grading criteria [2]. Building evidence suggests that H3 K27M-mutant gliomas in adults represent a heterogeneous disease, and thus the prognostic significance of H3 K27M depends on the radiographic and histopathologic context [14–16]. Thus, despite the low grade histology in this case, it must be acknowledged that the patient did appear to have interval radiographic progression within the first 5 months after diagnosis, which prompted the initial resection.

In terms of low grade gliomas, the relative incidence of H3 K27M mutation in spinal subependymoma remains unknown. There have been four cases of subependymomas with the H3 K27M mutation reported in literature, and all were located in the brainstem [16]. More specifically, in this case series of 24 histopathologically confirmed subependymomas, 4 were positive for the H3 K27M mutation by immunostaining—confirmed via sanger sequencing—and all were located in the brainstem. All reported tumors had a KI67 labeling index of under 5%, as was also seen in both the primary and recurrent samples studied from our case. Interestingly, despite the detection of H3 K27M mutation, these four patients displayed a slow tumor progression with a mean follow-up time of 3.5 years after the surgical resection. Likewise, our patient has been followed 47 months after the 1st resection and pathology from the second resection showed histopathological features of low grade glioma similar to the first resection. The global methylation pattern seen in this patient’s tumor was consistent with the presence of the H3 K27M mutation which is likely a major driver of this tumor’s epigenetic features.

Given the presence of Rosenthal fibers in the recurrent surgical sample, a histological diagnosis of pilocytic astrocytoma was also considered. Notably, pilocytic astrocytomas have also been infrequently associated with H3 K27M mutation [2, 15–17]. However, given that Rosenthal fibers were seen only focally in the recurrent sample, coupled with the fact that the areas with Rosenthal fibers were negative for the H3 K27M immunostain, we interpret this finding to most likely represent reactive piloid gliosis near the prior surgical resection. Furthermore, pilocytic astrocytomas are generally diffusely positive for OLIG2 and SOX10 [18, 19], whereas in this case OLIG2 and SOX10 expression was seen in only a subset of cells. In fact, the low levels of OLIG2 and SOX10 immunostaining seen in this tumor are more consistent with what has been reported for subepenymomas [12, 18].

Somatic *PTPN11* mutations are relatively infrequent in low grade glial neoplasms and have not been previously reported in subependymomas. There is one case in the literature describing an intraventricular subependymoma in a patient with Noonan Syndrome and a germline mutation in *PTPN11* [20]. Of note, our patient did not have any signs/symptoms, nor an existing clinical diagnosis, of Noonan syndrome, though genomic sequencing of peripheral blood was not performed.

In both the previously published case as well as in our presented case, it is unclear if the *PTPN11* mutation is a driver alteration linked to the emergence of the neoplasm, or if it is a passenger mutation. There is one report of high grade brainstem glioma harboring both *PTPN11* and *H3F3A* mutations, as well as one patient in a cohort of pediatric glioma with co-occurrence of H3 K27M mutations and other alterations in the MAPK pathway, including in *BRAF* and *FGFR1* [7, 21]. However, to our knowledge, this is the first case of low grade glial neoplasm of the spinal cord harboring both H3 K27M and *PTPN11* mutations. It is possible that this tumor is part of a unique subset of low grade gliomas with paired alterations in H3 K27M and the MAPK pathway, and makes the case for inclusion of these variants in the workup of histomorphologically low grade glial neoplasms. While the patient described here survived five years to date since diagnosis, the prognosis of such a mutation combination in this setting is still poorly understood and requires further investigation.

## Conclusion

We present the first reported case of a low grade thoracic spinal glioma harboring both H3 K27M and PTPN11 mutations. The tumor is slow growing, but showed some radiographic progression over the course of 3 years. Histopathological analysis of both the primary and recurrent tumor demonstrated a cytologically bland glial neoplasm with a low proliferation index. Interestingly, DNA methylation analysis demonstrated that the tumor clustered with H3 K27M gliomas and MYB low grade gliomas. Thus, the prognostic significance of co-occurring H3 K27M and *PTPN11* mutations is poorly understood in this context and merits further investigation. As building evidence suggests H3 K27M-mutant gliomas with concurrent *BRAF* mutations behave less aggressively than those with isolated H3 K27M mutations [14], this case may represent a unique subset of LGG with paired H3 K27M and MAPK alterations. Further, this case highlights the potential utility of long-term follow-up of patients with low grade tumors and prognostically worrisome genetic variants.

## Supplementary Information

**Additional file 1: Figure 1**. Pre-operative imaging and intraoperative images of second resection. (A) Sagittal T1 post-contrast MRI at 32 months demonstrating new contrast enhancement (B) Debulking of recurrent intramedullary tumor with no clear margins from spinal cord. (C) Extensive tumor debulking with improved mass effect on the spinal cord.

## Data Availability

Data available upon request.

## Abbreviations

H3 K27M: Amino acid change of lysine to methionine at the 28th amino acid (p.K28M) in H3.1 or H3.3 proteins
PTPN11: Protein tyrosine phosphatase, non-receptor type 11
MRI: Magnetic Resonance Imaging
WHO: World Health Organization
LGG: Low grade glioma
