# Supplementary figures and images for: Thoracic low grade glial neoplasm with concurrent H3 K27M and PTPN11 mutations
Source: Acta Neuropathol Commun. 2022 Apr 28;10:64. doi: 10.1186/s40478-022-01340-9 (PMC9052613; doi:10.1186/s40478-022-01340-9)

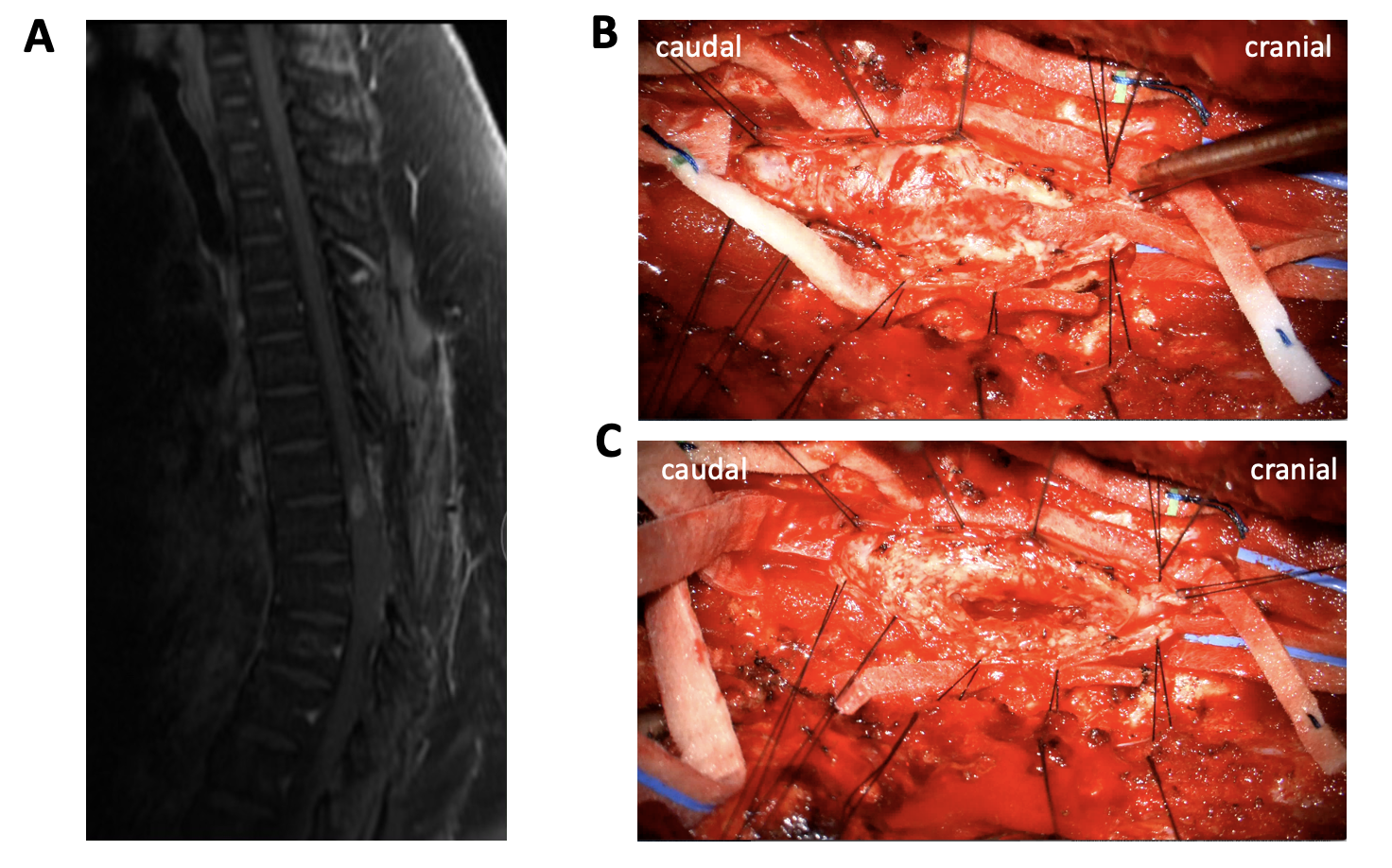

Supplement: Supplementary file 1 — Additional file 1: Figure 1. Pre-operative imaging and intraoperative images of second resection. (A) Sagittal T1 post-contrast MRI at 32 months demonstrating new contrast enhancement (B) Debulking of recurrent intramedullary tumor with no clear margins from spinal cord. (C) Extensive tumor debulking with improved mass effect on the spinal cord. [file 40478_2022_1340_MOESM1_ESM.png]
